# A seven-year disease-free survivor of malignant pleural mesothelioma treated with hyperthermia and chemotherapy: a case report

**DOI:** 10.1186/1752-1947-6-427

**Published:** 2012-12-28

**Authors:** Noriyuki Okonogi, Takeshi Ebara, Hitoshi Ishikawa, Daisaku Yoshida, Manabu Ueno, Toshitaka Maeno, Tatsuo Suga, Takashi Nakano

**Affiliations:** 1 Department of Radiation Oncology, Gunma University Graduate School of Medicine, 3-39-22 Showa-machi, Maebashi, Gunma, 371-8511, Japan; 2 Department of Radiation Oncology, Saitama International Medical Center, Saitama, Japan; 3 Department of Radiation Oncology, University of Tsukuba, Ibaraki, Japan; 4 Department of Medicine and Biological Science, Gunma University Graduate School of Medicine, Gunma, Japan

**Keywords:** Chemotherapy, Hyperthermia, Long-term survivor, Malignant pleural mesothelioma

## Abstract

**Introduction:**

Malignant pleural mesothelioma was once a rare finding but its incidence is increasing worldwide, most likely because of widespread exposure to asbestos. Although complete surgical resection is considered the only curative treatment, the results of surgery have shown a median survival time of only one year. In inoperable cases, chemotherapy, radiotherapy, and a combination of both have been considered as palliative therapy. Therefore, outcomes for inoperable cases have been poor. Here, we report the case of a long-term survivor treated with hyperthermia and chemotherapy.

**Case presentation:**

A 61-year-old Japanese man with a performance status of 1 due to chest pain was referred to our hospital. He had a history of asbestos exposure for approximately five years. A computed tomography scan showed diffuse extensive right pleural thickening with small nodular lesions, and video-assisted thoracoscopy revealed tumor invasion of the ipsilateral chest wall muscles. The histopathologic findings were consistent with a diagnosis of malignant pleural mesothelioma (sarcomatoid type). The tumor was diagnosed as being stage cT3N0M0. Our patient refused any invasive therapies including surgery and radiotherapy, and was therefore treated with hyperthermia and systemic chemotherapy with agents such as cisplatin and irinotecan. He underwent three hyperthermia sessions and a single course of chemotherapy without any severe complications. One month after treatment, a follow-up computed tomography scan showed no definitive abnormality in the thoracic space. Our patient has subsequently survived without any evident disease for more than seven years.

**Conclusions:**

The combination of hyperthermia and chemotherapy may be a novel and safe therapeutic option for malignant pleural mesothelioma, and can be considered for patients ineligible for radical treatment. Further clinical studies of the combination of hyperthermia and chemotherapy are needed to confirm the effects of this treatment on malignant pleural mesothelioma.

## Introduction

Malignant pleural mesothelioma (MPM) was once a rare finding, but its incidence is increasing worldwide, most likely because of widespread exposure to asbestos [1,2]. MPM is considered to have a poor prognosis, and complete surgical resection is the only curative treatment. However, the post-operative median survival time (MST) after surgery is only approximately one year [3-5]. Moreover, there is no standard treatment for inoperable MPM. We report a case of a seven-year disease-free survivor who was diagnosed as having MPM and was treated with hyperthermia and chemotherapy.

## Case presentation

**Figure 1:**
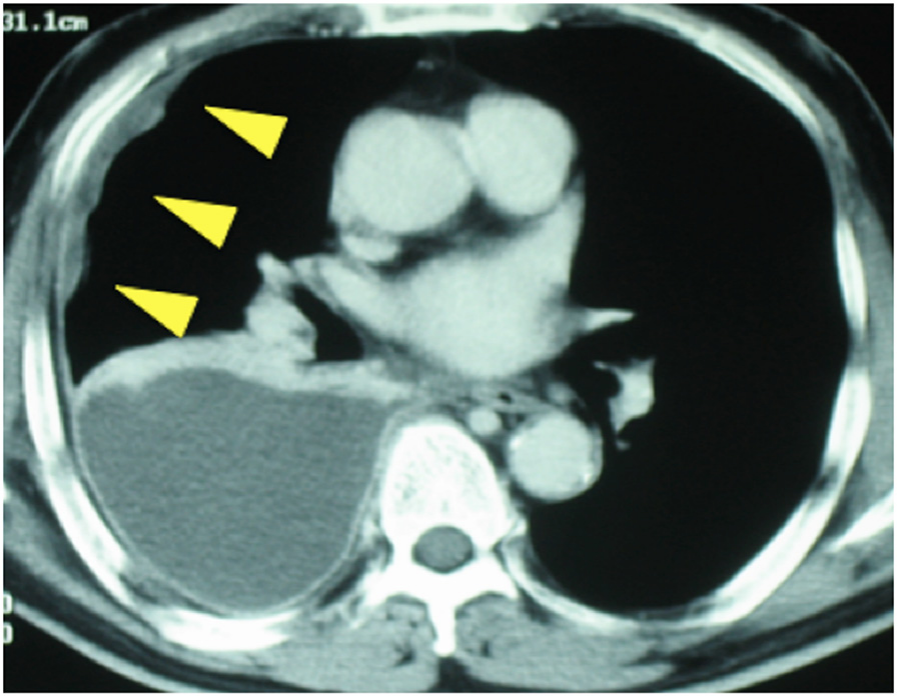
**Computed tomography images before treatment.** A chest computed tomography scan before treatment shows right pleural diffuse thickening (arrows).

A 61-year-old Japanese man with a performance status of 1 due to chest pain was referred to our hospital. He reported a persistent fever for approximately one month. The results of a computed tomography (CT) scan revealed diffuse extensive right pleural thickening with small nodular lesions (Figure 1). Our patient was a social drinker with a smoking history of 40 pack-years as well as a history of asbestos exposure for approximately five years.

**Figure 2:**
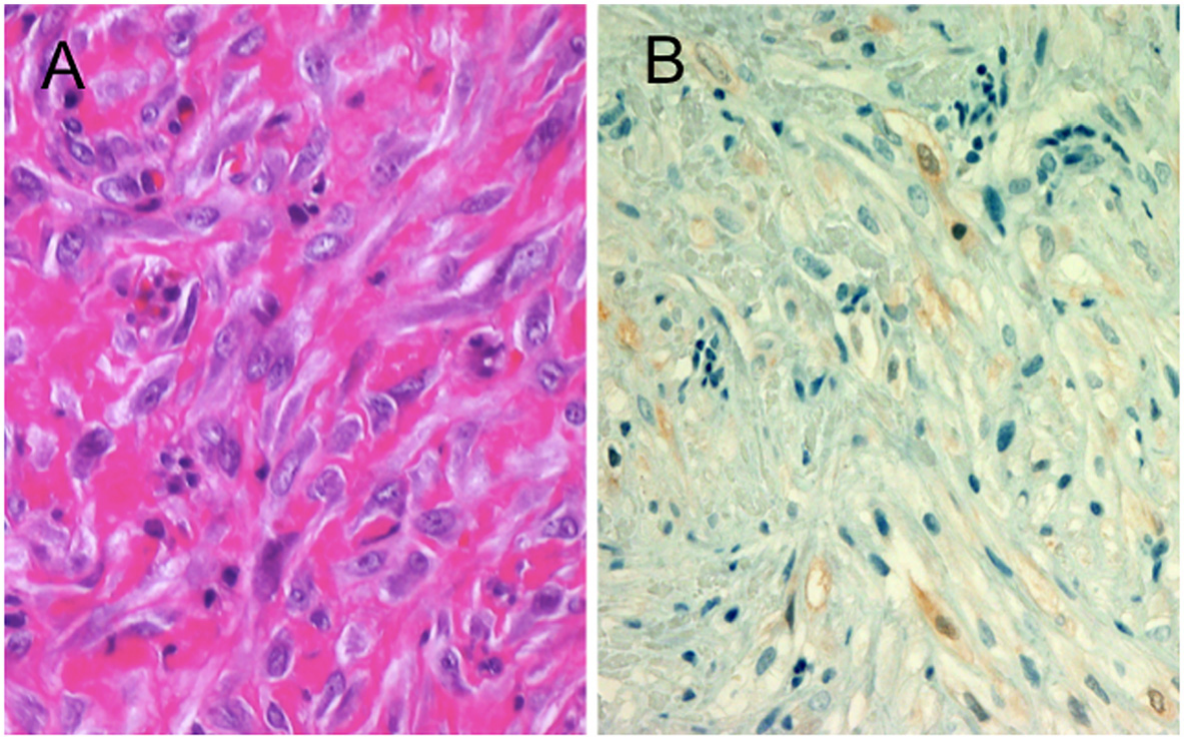
**Pathological findings. (A)** Disarrayed proliferation of large, spindle-shaped tumor cells in fibrous stroma was revealed by hematoxylin and eosin staining. (**B**) Positive staining of cells for calretinin.

To confirm the diagnosis of MPM, a video-assisted thoracoscopic lung biopsy was performed, which revealed tumor invasion of the ipsilateral chest wall muscles. Although the tumor markers for malignant mesothelioma, such as cytokeratin fragment (CYFRA) and tissue polypeptide antigen (TPA), were within normal limits, the hyaluronic acid concentration in the right pleural effusion was 55,000ng/mL, which was suggestive of MPM [3]. Hematoxylin and eosin staining of the specimen revealed disarrayed proliferation of large, spindle-shaped tumor cells with fibrous stroma (Figure 2). Immunohistochemistry testing was positive for epithelial membrane antigen, calretinin, vimentin, and cytokeratin 5/6. In contrast, staining results for carcinoembryonic antigen (CEA), tissue-specific transcription factor 1 (TTF-1), S-100, and CD34 were negative. These histopathologic findings were consistent with the diagnosis of MPM (sarcomatoid type). On the basis of these clinical examinations, the tumor was diagnosed as being T3N0M0 stage III MPM according to the Union for International Cancer Control guidelines.

Our patient was eligible for surgery; however, he refused any invasive therapies such as surgery and radiotherapy. Therefore, we proposed systemic chemotherapy with hyperthermia, which was used by our institution at that time for unresectable lung cancers with or without malignant pleural effusion [4,5]. Our patient agreed to this treatment strategy; he provided his written informed consent after a comprehensive discussion regarding the nature of his illness and standard therapeutic options, including surgery and combined modality therapy. Three weeks after the biopsy, systemic chemotherapy with hyperthermia was administered once a week. The chemotherapy regimen included cisplatin 60mg/m^2^ (100mg/body) on day one and irinotecan 60mg/m^2^ (100mg/body) on days one, eight, and 15. Hyperthermia was performed using a radiofrequency (RF)-capacitive heating apparatus (Thermotron-RF 8; Yamamoto Vinita Co. Ltd., Osaka, Japan) once a week (on days one, eight, and 15) for approximately 60 minutes immediately after irinotecan administration. The electrodes measured 30cm in diameter and were placed in front of and behind our patient to heat the entire thoracic cavity. A pair of overlay boluses were used to prevent any edge effect and for cooling the surface of the body. Thermosimulation revealed that the nodular lesions were approximately 42.5°C and the right pleural thickening >40°C.

**Figure 3:**
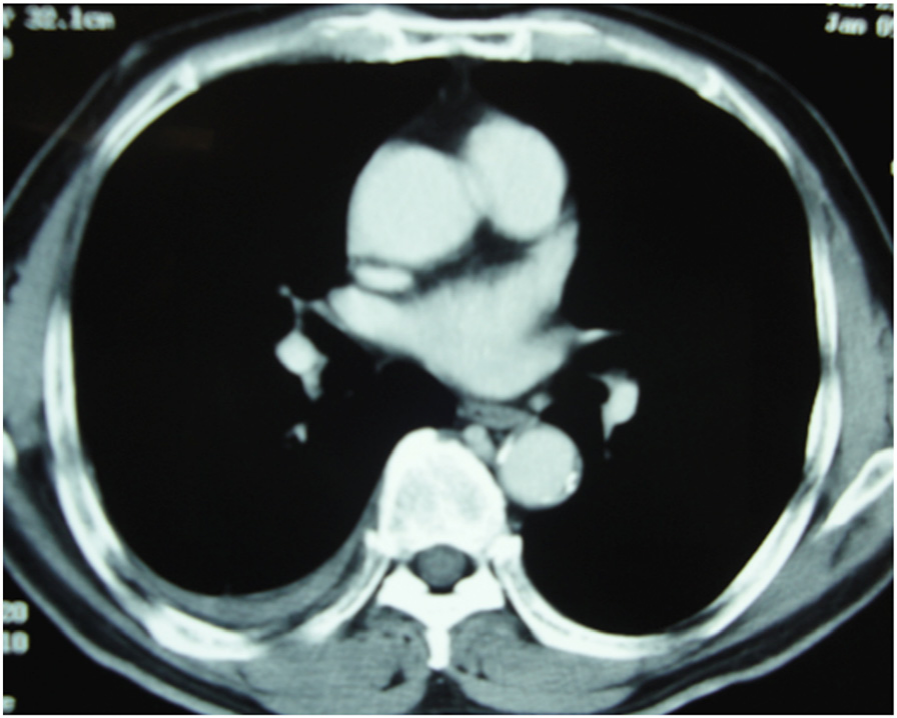
**A computed tomography image at the time of complete response.** One month after the treatment completion, the pleural thickening had completely disappeared. A small amount of right pleural effusion was observed, but has not increased since.

**Figure 4:**
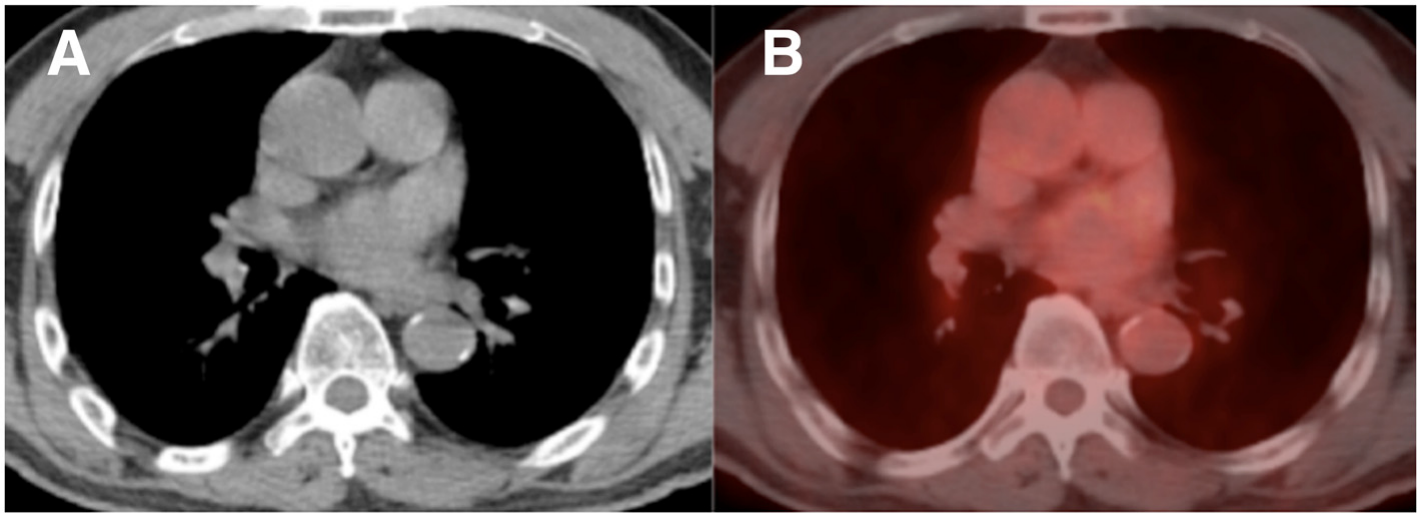
**Computed tomography and positron emission tomography-computed tomography images six years after treatment.** A chest computed tomography scan **(A)** and a positron emission tomography scan **(B)** were obtained six years after our patient’s completion of treatment. The diffuse pleural thickening has disappeared. There is no evidence of recurrence or metastasis.

Shortly before initiating the second chemotherapy course, a month after the first treatment cycle, a CT scan revealed that the pleural thickening had disappeared (Figure 3). The right pleural effusion was drained only once during biopsy, and pleural effusion had not increased thereafter. Consequently, our patient underwent three sessions of hyperthermia and a single course of chemotherapy without any severe complications. He was subsequently followed-up by CT scan and 18F-fluorodeoxy-glucose-positron emission tomography (18FDG-PET), performed six years after the end of treatment; the results revealed no evidence of recurrence or metastasis (Figure 4). Our patient has been disease free for more than seven years without any complications.

## Discussion

MPM develops after asbestos exposure and has a long latency period. Its incidence is currently increasing worldwide because of the extent of asbestos exposure between the years 1965 and 1980 [1,2]. MPM has a poor prognosis, mainly because of its inherent resistance to conventional treatment. Therefore, it is necessary to identify novel treatment strategies.

Radical surgical resection is considered the only curative treatment; however, the post-operative MST for such patients is only approximately 12 months. The benefits of such an aggressive approach have been questioned because the treatment-related mortality rate is 7.0 percent to 11.5 percent [6-8]. Recently, trimodality therapies such as combinations of surgical resection and chemoradiotherapy for operable MPM cases have improved the prognosis [9-11]. Flores *et al*. reported a MST of 19 months for operable MPM [11], and Perrot *et al*. reported a five-year overall survival rate of 10 percent following trimodality treatment. However, the rate of treatment-related mortality remains high, and is equivalent to approximately 5 percent of all patients who undergo trimodality treatments [9,10]. Additionally, the rate of treatment-related morbidities such as atrial arrhythmias, respiratory failure, and pneumonia, among others, is 25 percent to 55 percent [9-11]. These complications can reduce a patient’s quality of life.

Moreover, chemotherapy, radiotherapy, or a combination of both have been considered as palliative therapy in inoperable cases. Over the past two decades, a number of single-agent and combination chemotherapy studies have been performed for MPM. Unfortunately, all the published reviews have reported poor response rates of approximately 30 percent, and a short MST of approximately 10 months [12,13]. In a recent study, Vogelzang *et al*. reported that MST for subjects in the pemetrexed/cisplatin arm was 12.1 months, whereas that for subjects in the cisplatin monotherapy arm was 9.3 months [14]. However, this result is still not a satisfactory one.

In our patient’s case, he refused any invasive therapy and thus underwent systemic chemotherapy with hyperthermia. The chemotherapeutic doses of cisplatin 60mg/m^2^ on day one and irinotecan 60mg/m^2^ on days one, eight, and 15, were relatively low compared with the conventional doses (Table 1) [15-18]. Therefore, we speculated that the remarkable efficacy may have been due to an intrinsic effect of hyperthermia or a synergistic effect of the hyperthermia-chemotherapy combination.

**Table 1:** Cisplatin-based conventional chemotherapy regimens and their efficacy against malignant pleural mesothelioma

**Lead author/reference**	**Chemotherapy regimen**	**Admissions interval (median courses)**	**Number of patients**	**Response rate**	**Median survival (months)**
Vogelzang [14]	Cisplatin (75mg/m^2^ on day one)/pemetrexed (500mg/m^2^ on day one)	Every three weeks, 6.0 courses	226	41 percent	12.1
Nakano [15]	Cisplatin (60mg/m^2^ on day one)/irinotecan (60mg/m^2^ on days one, eight, and 15)	Every four weeks, 2.6 courses	15	27 percent	6.5
Tsavaris [16]	Cisplatin (100mg/m^2^ on day one)/vinblastine (6mg/m^2^ on days one and eight)	Every four weeks, 4.0 courses	20	25 percent	N/A
Ardizzoni [17]	Cisplatin (60mg/m^2^ on day 1)/doxorubicin (60mg/m^2^ on day one)	Every three to four weeks, 3.0 to 4.0 courses	26	25 percent	10
Nowak [18]	Cisplatin (100mg/m^2^ on day one)/gemcitabine (1000mg/m^2^ on days one, eight, and 15)	Every four weeks, 4.0 courses	53	33 percent	11.2
Present report	Cisplatin (60mg/m^2^ on day one)/irinotecan (60mg/m^2^ on days one, eight, and 15)	One course with hyperthermia		Complete response	>84

N/A, not applicable.

Hyperthermia is known to be directly cytotoxic to cancer cells [19-21]. When cells are exposed to elevated temperatures, damage is inflicted at multiple sites; the predominant molecular target appears to be proteins such as heat shock proteins (HSPs) [22]. Roth *et al*. reported the effects of heat stress on HSPs [23]. It was noted that hyperthermia induced the downregulation heat-stress-induced Hsp40 and Hsp70 expression and reduced the survival of mesothelioma cells.

Some clinical reports have also reported the benefits of hyperthermia. Matsuzaki *et al*. reported on intra-pleural chemotherapy perfusion with hyperthermia to induce MPM cell apoptosis. MPM cells obtained from pleural effusions showed apoptotic action, peaking at 24 hours after perfusion [24]. Xia *et al*. reported significant prolongation of MST by hyperthermia combined with intra-thoracic chemotherapy and radiotherapy for MPM. Although a complete response was not achieved, MST was 27.1 months without any severe side effects [25]. These reports suggest that hyperthermia may have a special effect on MPM cells, and that the combination of hyperthermia and chemotherapy may act synergistically.

## Conclusions

This report describes a rare case of a long-term survivor with MPM. Our patient has been disease-free for more than seven years without any complications. The combination of hyperthermia and chemotherapy may be a novel and safe therapeutic option for MPM and can be considered in cases ineligible for radical treatment. Further clinical studies of the hyperthermia-chemotherapy combination are required to confirm its effects on MPM.

## Consent

Written informed consent was obtained from the patient for publication of this case report and any accompanying images. A copy of the consent form is available for review by the Editor-in-Chief of this journal.

## Competing interests

The authors declare that they have no competing interests.

## Authors’ contributions

Data from our patient were collected by NO and DY. The hyperthermia sessions were performed by HI. Chemotherapy was administered by MU, TM, and TS. The manuscript was prepared by NO. Corrections and/or improvements were suggested by TE and TS. Major revisions were made by TE and TN. All authors read and approved the final manuscript.
